# Supplementary material for: Downregulation of the enhancer of zeste homolog 1 transcriptional factor predicts poor prognosis of triple-negative breast cancer patients
Source: PeerJ. 2022 Jul 12;10:e13708. doi: 10.7717/peerj.13708 (PMC9285492; doi:10.7717/peerj.13708)
Supplement: Supplemental Information 3 [file peerj-10-13708-s003.docx]

**Supplementary Table 2. A negative correlation was found between EZH1 expression and *CCNA2*, *CCNB1*, *MAD2L1*, and *PKMYT1* expression.**

| Gene 1 | Gene 2 | Pearson' r | P |  |  | Gene 1 | Gene 2 | Pearson' r | P |
| --- | --- | --- | --- | --- | --- | --- | --- | --- | --- |
| EZH1 | CCNB1 | -0.455 | <0.0001 |  |  | EZH1 | CCNA2 | -0.365 | <0.0001 |
| EZH1 | CCNA2 | -0.448 | <0.0001 |  |  | EZH1 | MAD2L1 | -0.389 | <0.0001 |
| EZH1 | CCNB1 | -0.308 | <0.0001 |  |  | EZH1 | MAD2L1 | -0.379 | <0.0001 |
| EZH1 | MAD2L1 | -0.322 | <0.0001 |  |  | EZH1 | PKMYT1 | -0.28 | <0.0001 |
| EZH1 | MAD2L1 | -0.403 | <0.0001 |  |  | EZH1 | CCNA2 | -0.322 | <0.0001 |
| EZH1 | PKMYT1 | -0.344 | <0.0001 |  |  | EZH1 | CCNA2 | -0.232 | <0.0001 |
| EZH1 | MAD2L1 | -0.245 | <0.0001 |  |  | EZH1 | CCNB1 | -0.189 | 0.0001 |
| EZH1 | CCNB1 | -0.637 | <0.0001 |  |  | EZH1 | CCNA2 | -0.236 | 0.0017 |
| EZH1 | CCNA2 | -0.559 | <0.0001 |  |  | EZH1 | CCNA2 | -0.238 | 0.0034 |
| EZH1 | PKMYT1 | -0.542 | <0.0001 |  |  | EZH1 | CCNB1 | -0.203 | 0.0066 |
| EZH1 | MAD2L1 | -0.6 | <0.0001 |  |  | EZH1 | MAD2L1 | -0.218 | 0.0074 |
| EZH1 | CCNB1 | -0.512 | <0.0001 |  |  | EZH1 | PKMYT1 | -0.145 | 0.0304 |
| EZH1 | CCNB1 | -0.54 | <0.0001 |  |  | EZH1 | PKMYT1 | -0.142 | 0.0330 |
| EZH1 | MAD2L1 | -0.513 | <0.0001 |  |  | EZH1 | PKMYT1 | 0.042 | 0.0546 |
| EZH1 | MAD2L1 | -0.46 | <0.0001 |  |  | EZH1 | MAD2L1 | -0.105 | 0.0743 |
| EZH1 | PKMYT1 | -0.495 | <0.0001 |  |  | EZH1 | PKMYT1 | -0.11 | 0.1481 |
| EZH1 | CCNA2 | -0.469 | <0.0001 |  |  | EZH1 | CCNB1 | 0.29 | 0.1502 |
| EZH1 | CCNA2 | -0.428 | <0.0001 |  |  | EZH1 | CCNA2 | -0.101 | 0.1794 |
| EZH1 | CCNB1 | -0.418 | <0.0001 |  |  | EZH1 | MAD2L1 | 0.05 | 0.3000 |
| EZH1 | CCNA2 | -0.167 | <0.0001 |  |  | EZH1 | MAD2L1 | 0.204 | 0.3165 |
| EZH1 | CCNB1 | -0.483 | <0.0001 |  |  | EZH1 | PKMYT1 | -0.018 | 0.4250 |
| EZH1 | CCNB1 | -0.428 | <0.0001 |  |  | EZH1 | CCNA2 | 0.131 | 0.5243 |
| EZH1 | MAD2L1 | -0.41 | <0.0001 |  |  | EZH1 | CCNB1 | -0.011 | 0.6405 |
| EZH1 | CCNB1 | -0.477 | <0.0001 |  |  | EZH1 | PKMYT1 | 0.088 | 0.6696 |
| EZH1 | PKMYT1 | -0.283 | <0.0001 |  |  |  |  |  |  |
