# Supplementary figures and images for: Downregulation of the enhancer of zeste homolog 1 transcriptional factor predicts poor prognosis of triple-negative breast cancer patients
Source: PeerJ. 2022 Jul 12;10:e13708. doi: 10.7717/peerj.13708 (PMC9285492; doi:10.7717/peerj.13708)

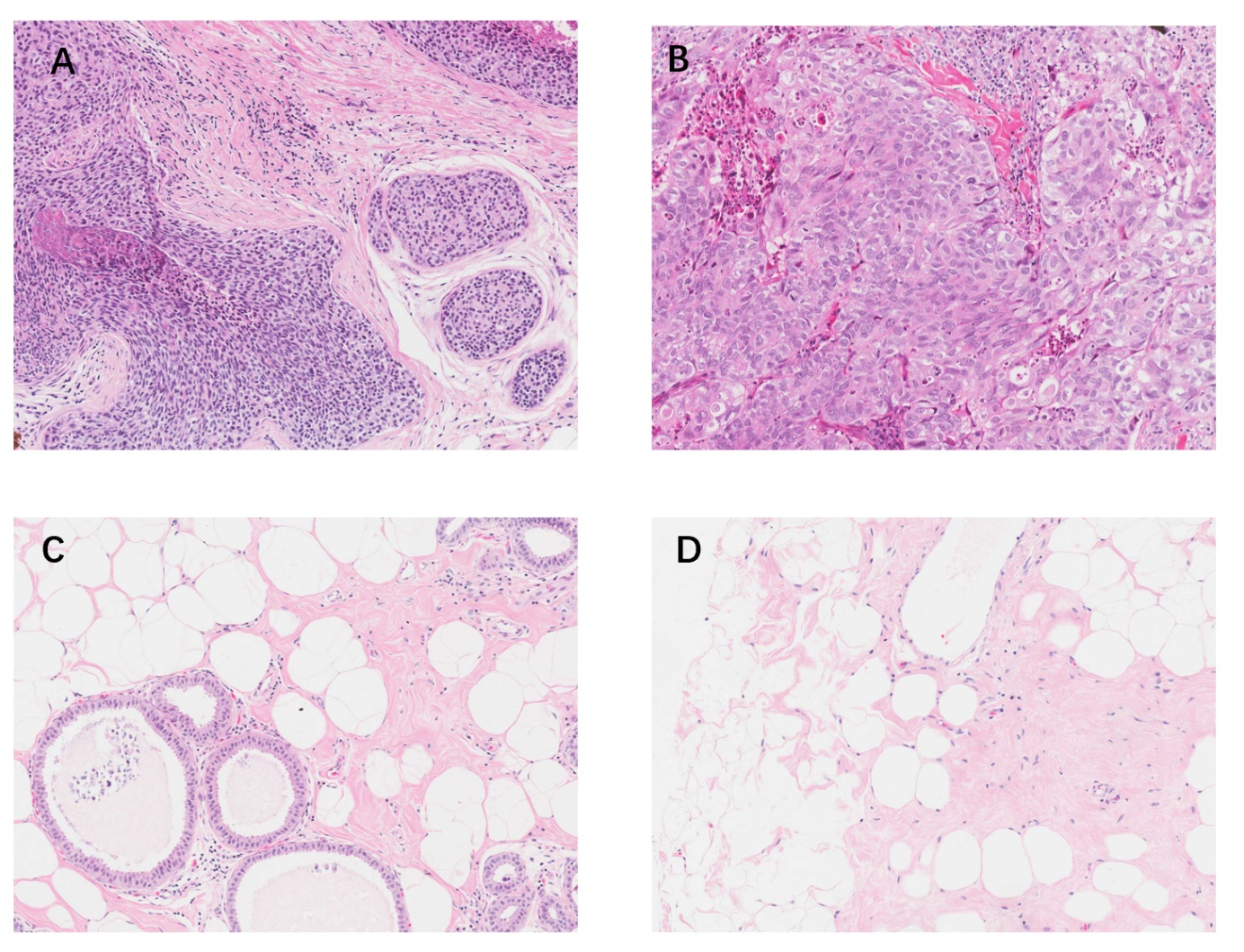

Supplement: Supplemental Information 4 — (A, B) HE staining of BC tissues. (C, D) HE staining of normal breast tissues. HE, hematoxylin and eosin. BC, breast cancer. [file peerj-10-13708-s004.png]

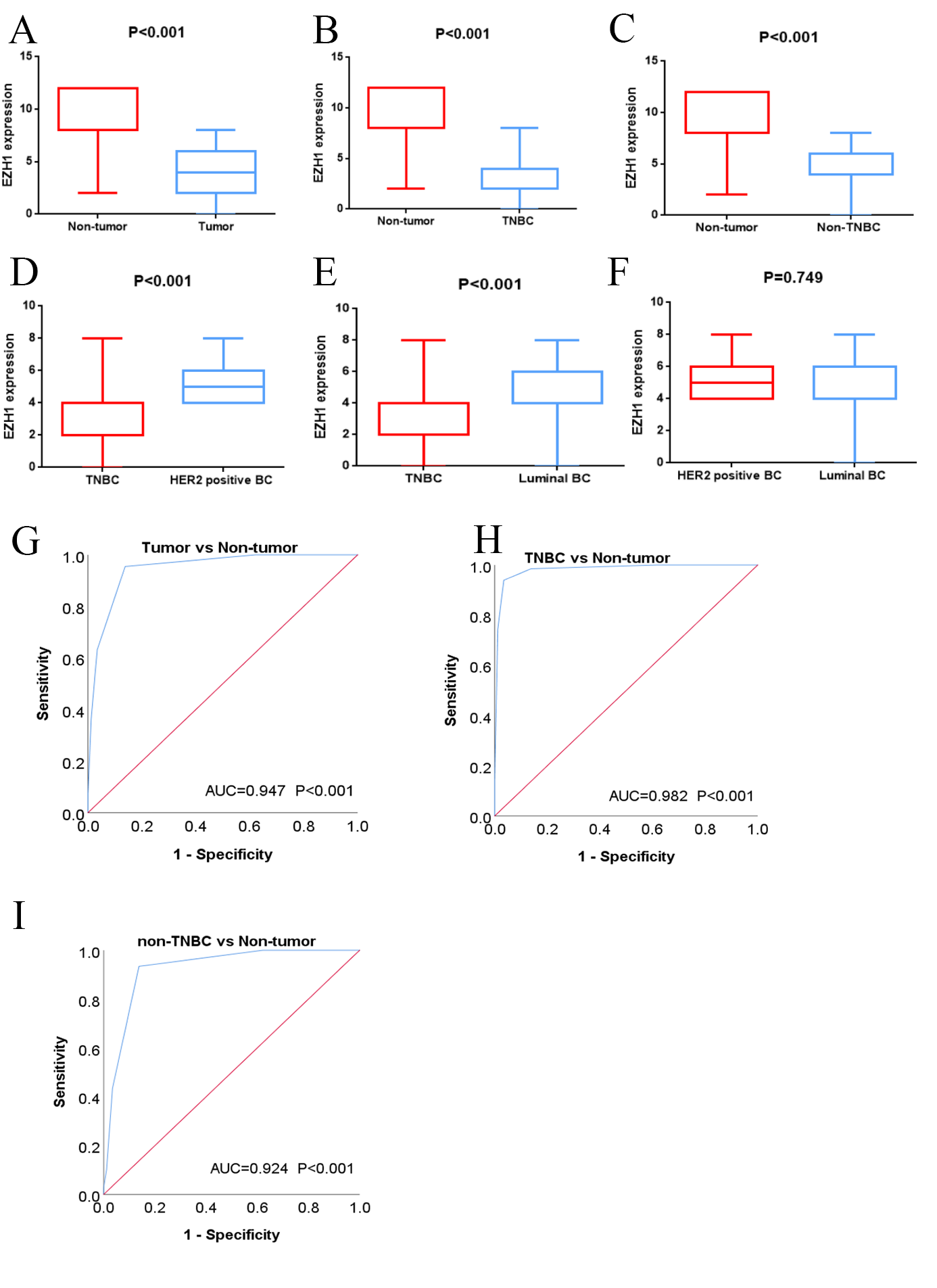

Supplement: Supplemental Information 5 — The protein expression of EZH1 in normal breast tissue was higher than in (A) BC, (B) TNBC, and (C) non-TNBC tissues. The protein expression of EZH1 in TNBC tissues was lower than in (D) HER2 + BC and (E) luminal BC tissues. (F) There was no significant difference in the protein expression of EZH1 between HER2 + BC and luminal BC tissues. EZH1 protein had a strong ability to differentiate (G) BC, (H) TNBC, and (I) non-TNBC tissues from normal breast tissues. BC, breast cancer; TNBC, triple-negative breast cancer. [file peerj-10-13708-s005.png]

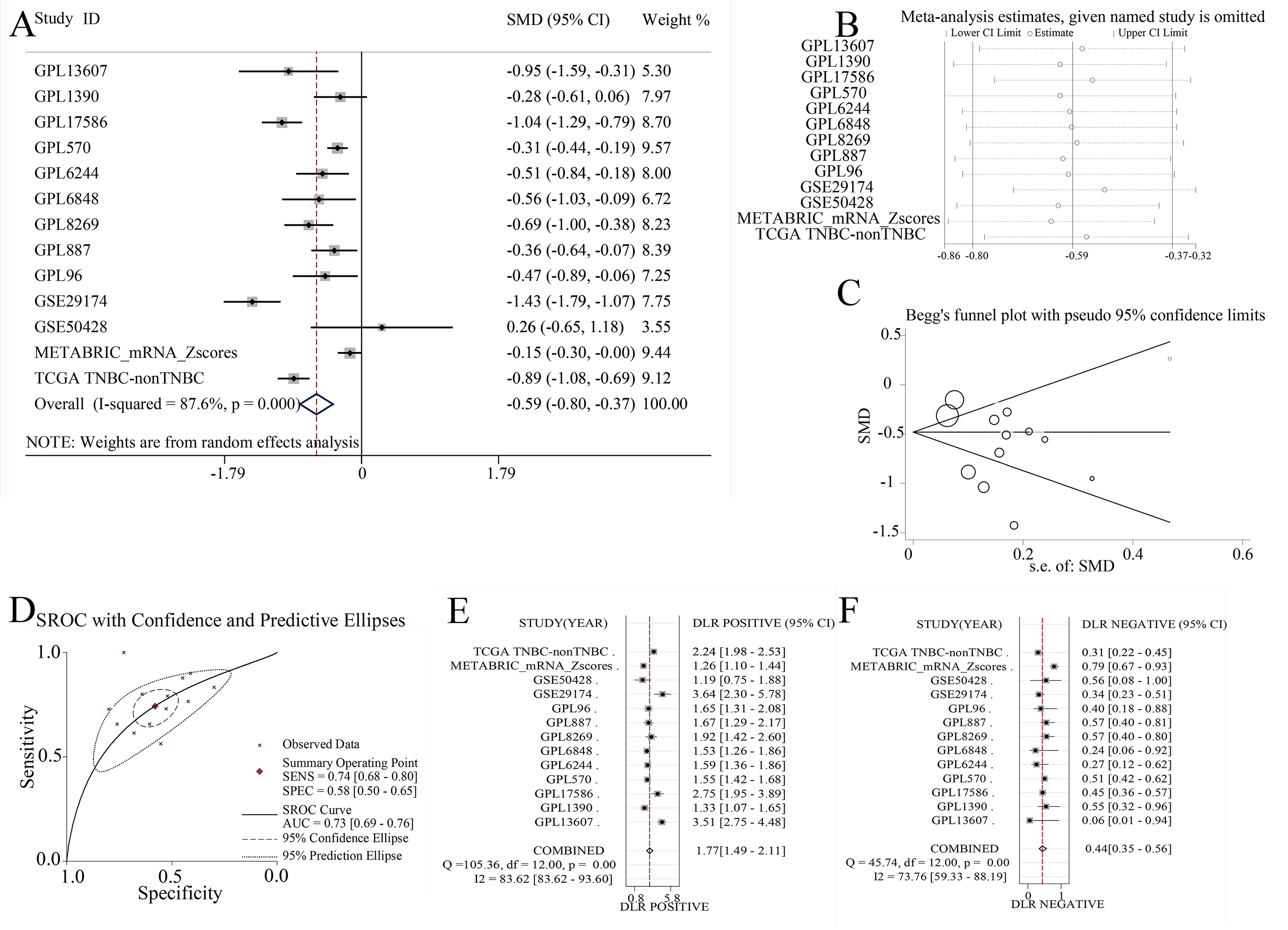

Supplement: Supplemental Information 6 — (A) EZH1 was significantly downregulated in TNBC tissues (SMD <0), and no obvious (B) heterogeneity or (C) publication bias was identified (Begg’s test: continuity corrected P = 0.669; Egger’s test: P = 0.224). (D) EZH1 had a moderate discriminatory ability between TNBC and non-TNBC tissues (area under the curve >0.7). The overall accuracy of EZH1 in discriminating TNBC from non-TNBC tissues were explored using (E) positive likelihood ratio forest plot and (F) negative likelihood ratio forest plot. [file peerj-10-13708-s006.png]

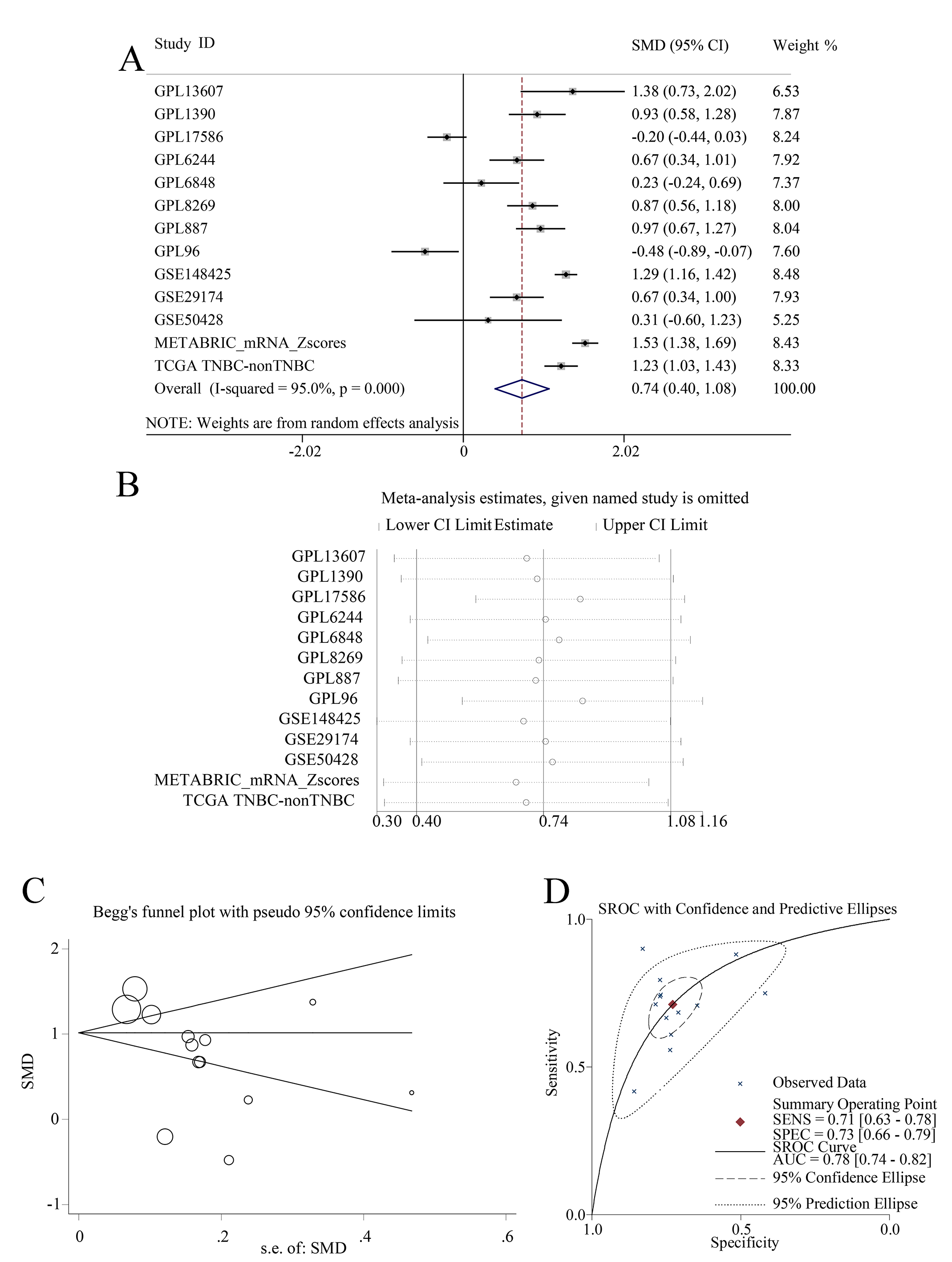

Supplement: Supplemental Information 7 — Unlike EZH1, (A) EZH2 was significantly upregulated in TNBC tissues (SMD >0), without obvious (B) heterogeneity or (C) publication bias (Begg’s test: continuity corrected P = 0.127; Egger’s test: P = 0.053). (D) EZH2 had a moderate discriminatory ability between TNBC and non-TNBC tissues (area under the curve >0.7). TNBC, triple-negative breast cancer. [file peerj-10-13708-s007.png]

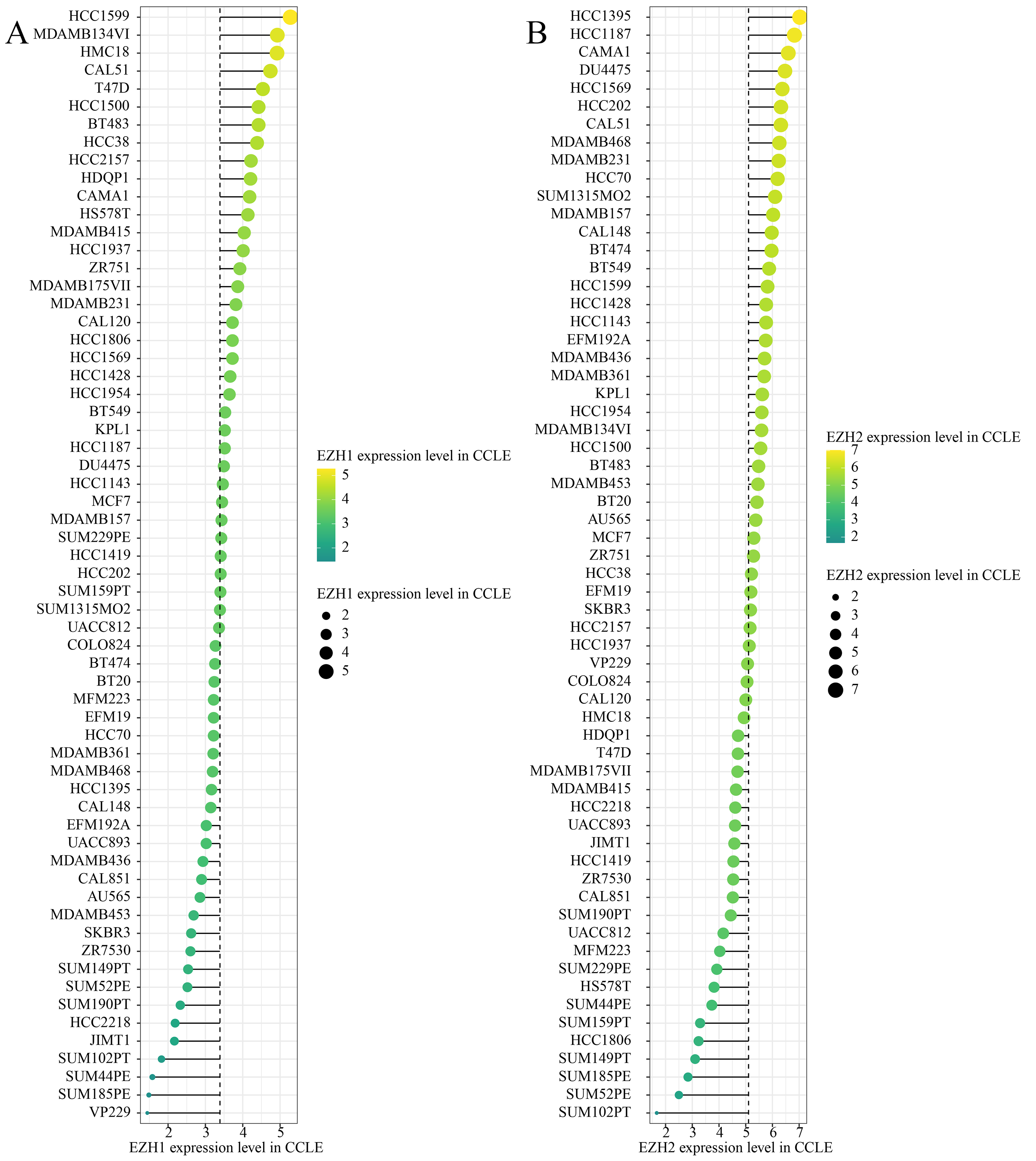

Supplement: Supplemental Information 8 — EZH1/EZH2 expression were validated in breast cancer cell lines using Cancer Cell Line Encyclopedia. (A) EZH1 (B) EZH2. [file peerj-10-13708-s008.png]

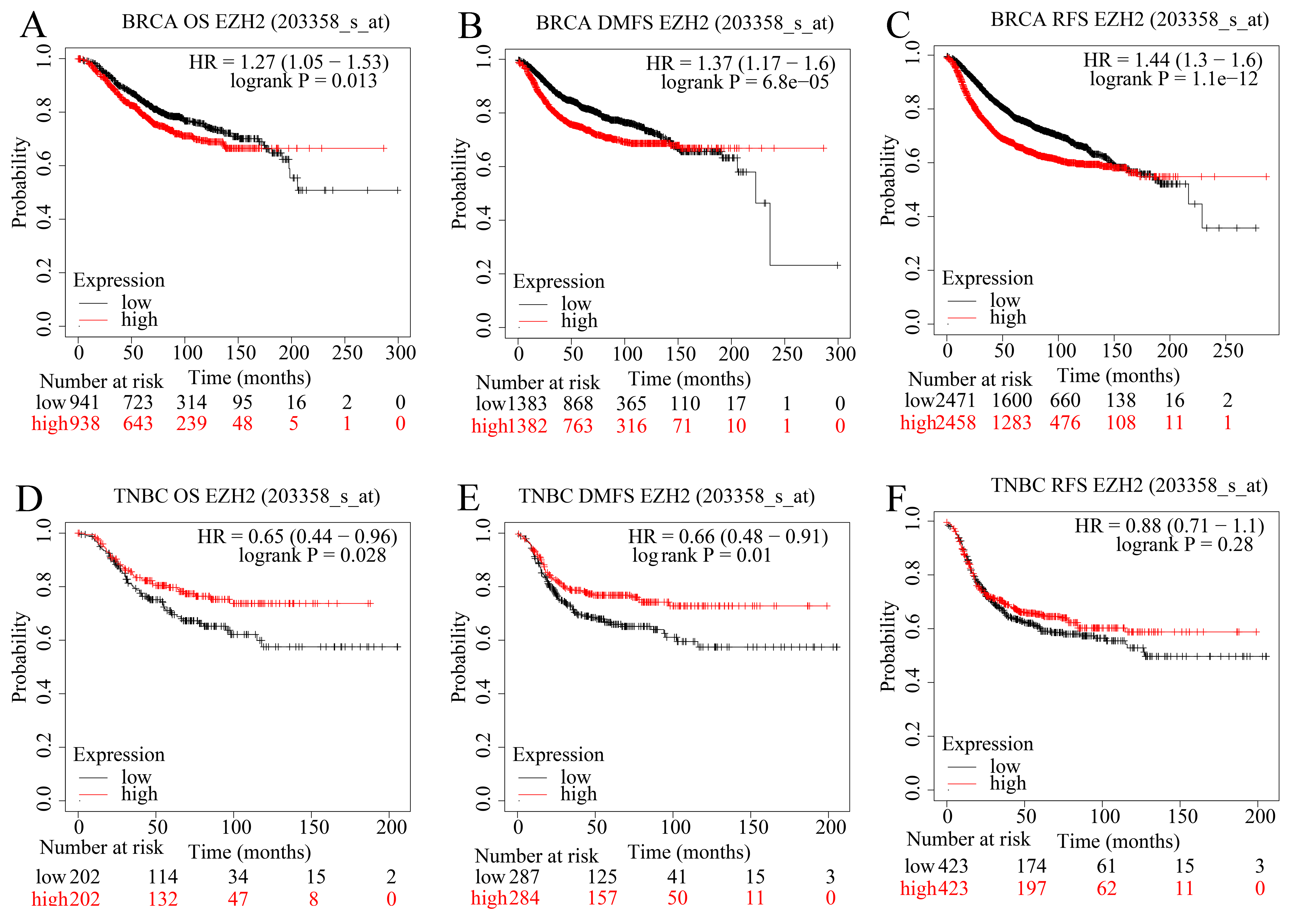

Supplement: Supplemental Information 9 — Higher EZH2 expression was significantly correlated with worse (A) OS, (B) DMFS, and (C) RFS in BC patients. TNBC patients with lower EZH2 expression were likely to exhibit poorer (D) OS, (E) DMFS, and (F) PFS. TNBC, triple-negative breast cancer; OS, overall survival; DMFS, distal metastasis-free survival; PFS, prognosis-free survival. [file peerj-10-13708-s009.png]

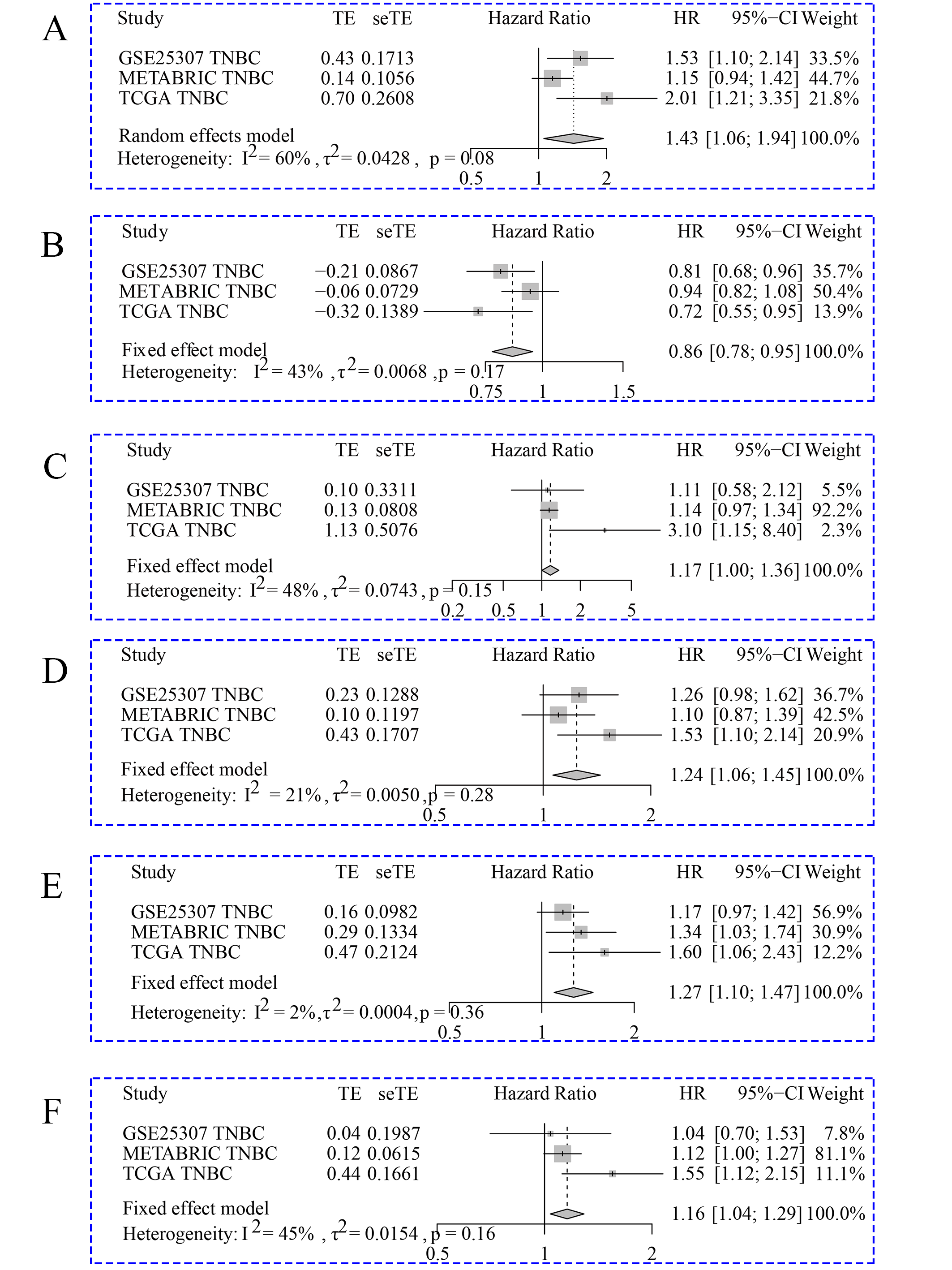

Supplement: Supplemental Information 10 — A: AKAP12. B: FABP7. C: GOLGA1. D: ITGB3. E: MATN3. F: PCDHB5. [file peerj-10-13708-s010.png]

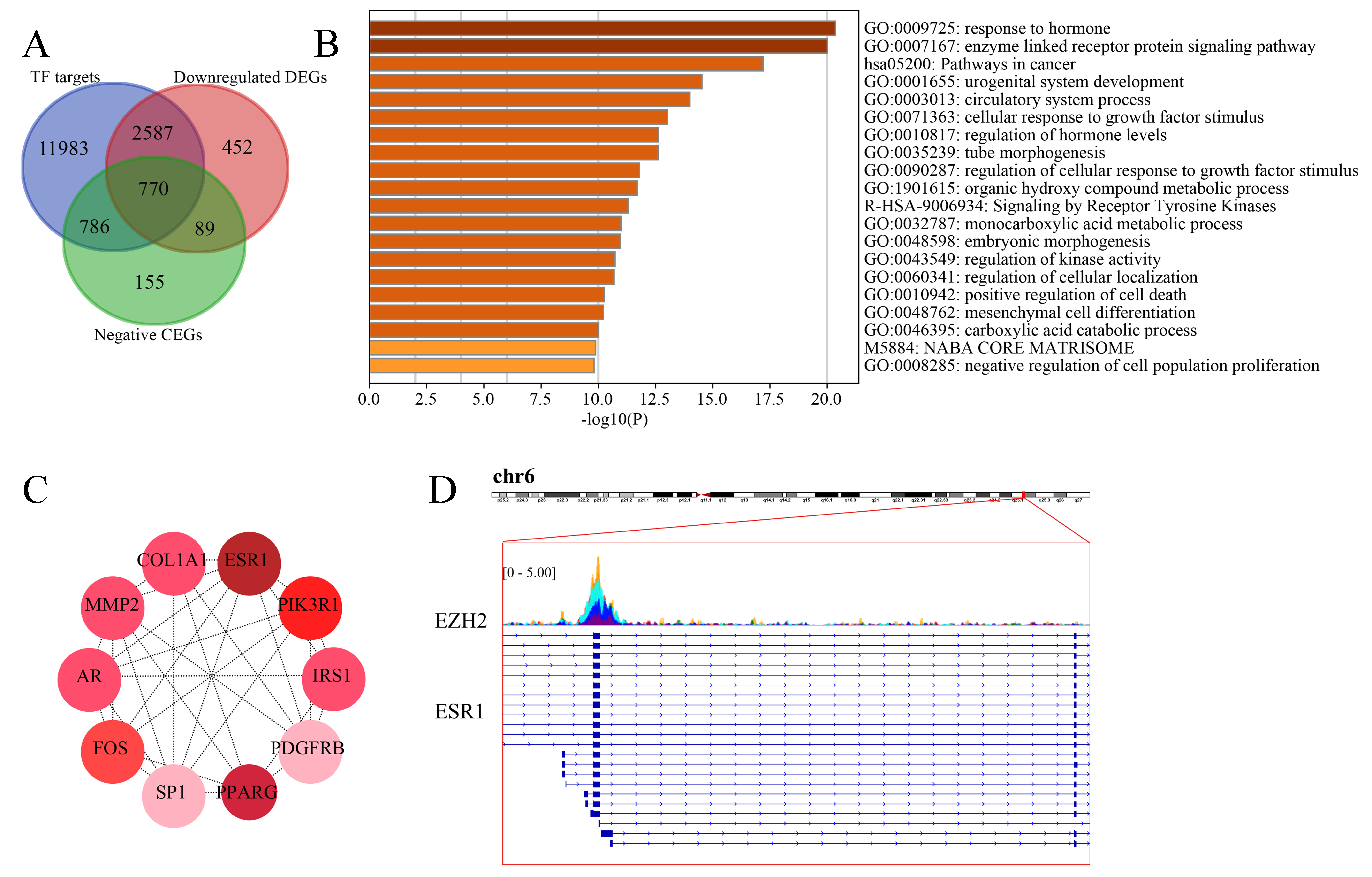

Supplement: Supplemental Information 11 — (A) Since EZH2 was involved in the transcriptional repression of its targets, we intersected the putative EZH2 targets, downregulated TNBC differentially expressed genes, and EZH2 negative co-expressed genes, where a total of 770 targets were identified. (B) The downregulated EZH2 transcriptional targets were enriched in response to hormone. (C) ESR1 was identified as a key gene in response to hormone. (D) The transcriptional factor binding sites for EZH2 and ESR1 was predicted. TNBC, triple-negative breast cancer. [file peerj-10-13708-s011.png]

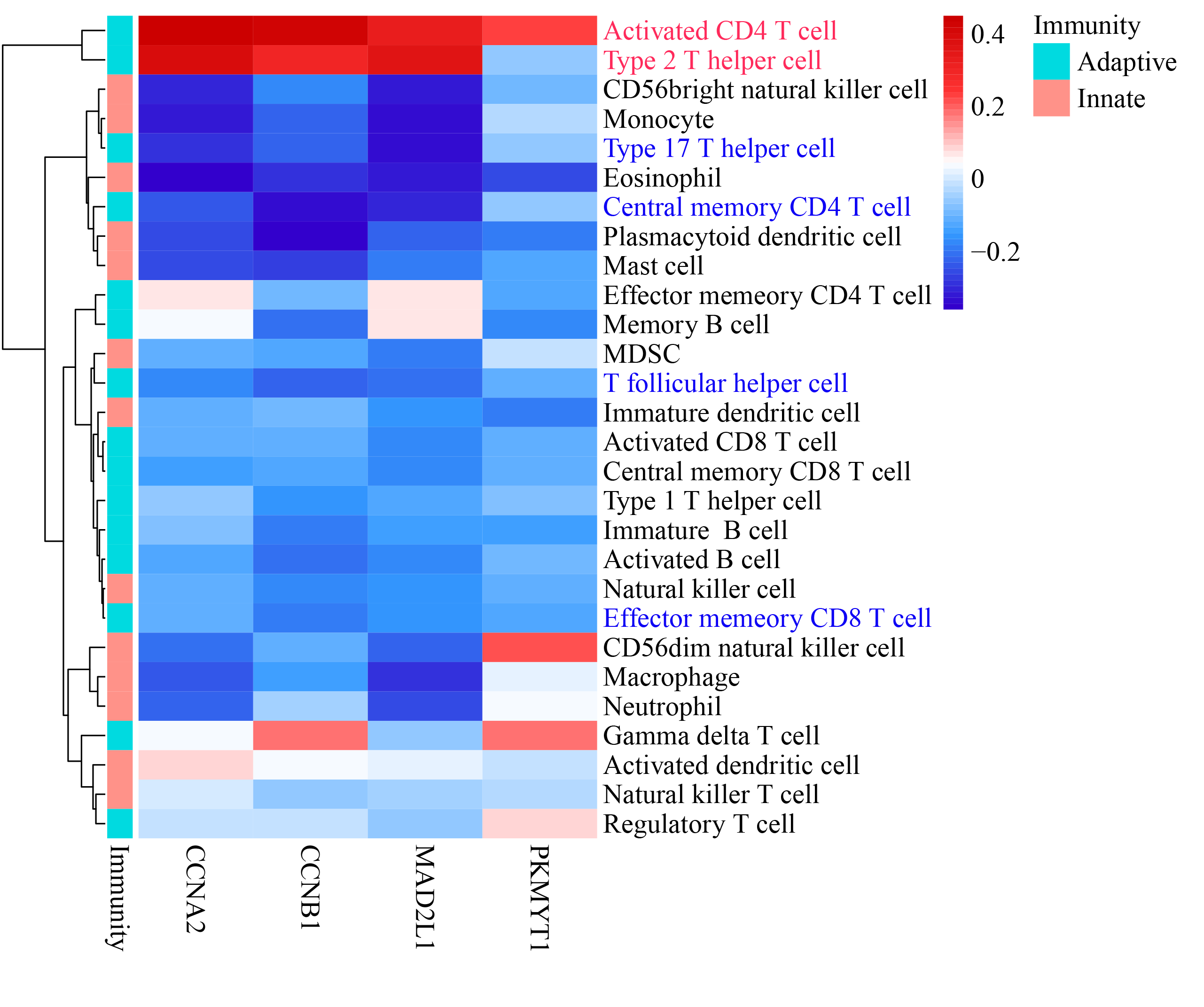

Supplement: Supplemental Information 12 — CCNB1 expression level was negatively correlated to the infiltration levels of central memory CD4+ T cell, type 17 T helper cell, and T follicular helper cell. However, a significantly positive correlation could be found between the expression level of CCNB1 and the infiltration levels of activated CD4+ T cell and type 2 T helper cell. [file peerj-10-13708-s012.png]
